# Radiomics analysis using MR imaging of subchondral bone for identification of knee osteoarthritis

**DOI:** 10.1186/s13018-022-03314-y

**Published:** 2022-09-14

**Authors:** Zhihao Xue, Liao Wang, Qi Sun, Jia Xu, Ying Liu, Songtao Ai, Lichi Zhang, Chenglei Liu

**Affiliations:** 1 Department of Radiology, Shanghai Ninth People’s Hospital, Shanghai Jiao Tong University School of Medicine, Zhizaoju road 639#, Huangpu District, Shanghai, 200011 China; 2 Institute for Medical Imaging Technology, School of Biomedical Engineering, Shanghai Jiao Tong University, Shanghai, China; 3 Shanghai Key Laboratory of Orthopaedic Implants, Department of Orthopaedic Surgery, Shanghai Ninth People’s Hospital, Shanghai Jiao Tong University School of Medicine, Shanghai, China; 4 Department of Orthopedic Surgery, Shanghai Jiao Tong University Affiliated Sixth People’s Hospital, 600 Yishan Road, Xuhui District, Shanghai, 200233 China; 5 Department of Radiology, Peking University Third Hospital, Beijing, China

**Keywords:** Magnetic resonance imaging, Knee osteoarthritis, Radiomics, Subchondral bone

## Abstract

**Background:**

To develop a magnetic resonance imaging (MRI)-based radiomics predictive model for the identification of knee osteoarthritis (OA), based on the tibial and femoral subchondral bone, and compare with the trabecular structural parameter-based model.

**Methods:**

Eighty-eight consecutive knees were scanned with 3T MRI and scored using MRI osteoarthritis Knee Scores (MOAKS), in which 56 knees were diagnosed to have OA. The modality of sagittal three-dimensional balanced fast-field echo sequence (3D BFFE) was used to image the subchondral bone. Four trabecular structural parameters (bone volume fraction [BV/TV], trabecular thickness [Tb.Th], trabecular separation [Tb.Sp], and trabecular number) and 93 radiomics features were extracted from four regions of the lateral and medial aspects of the femur condyle and tibial plateau. Least absolute shrinkage and selection operator (LASSO) was used for feature selection. Machine learning-based support vector machine models were constructed to identify knee OA. The performance of the models was assessed by area under the curve (AUC) of the receiver operator characteristic (ROC). The correlation between radiomics features and trabecular structural parameters was analyzed using Pearson’s correlation coefficient.

**Results:**

Our radiomics-based classification model achieved the AUC score of 0.961 (95% confidence interval [CI], 0.912–1.000) when distinguishing between normal and knee OA, which was higher than that of the trabecular parameter-based model (AUC, 0.873; 95% CI, 0.788–0.957). The first-order, texture, and Laplacian of Gaussian-based radiomics features correlated positively with Tb.Th and BV/TV, but negatively with Tb.Sp (*P* < 0.05).

**Conclusions:**

Our results suggested that our MRI-based radiomics models can be used as biomarkers for the classification of OA and are superior to the conventional structural parameter-based model.

**Supplementary Information:**

The online version contains supplementary material available at 10.1186/s13018-022-03314-y.

## Introduction

Osteoarthritis (OA) is the most common age-related degenerative joint disorder that causes joint pain and disability [1]. Currently, efficacious disease-modifying treatments for OA are still lacking due to its unclear pathological mechanism. Most of the research interest in the past has focused on cartilage degeneration. OA is a whole-joint disease involving the cartilage, subchondral bone, and synovium [2]. Particularly, the importance of subchondral bone in OA onset and progression has been well established [3, 4]. Subchondral bone alterations occurred in parallel with or predated cartilage loss in preclinical experimental studies [5]. Bone marrow edema, like bone marrow lesions (BML), is associated with pain in OA patients [6]. Furthermore, bone-targeted OA therapies improve OA symptoms or slow OA bone change progression[7]. Therefore, sensitive biomarkers of subchondral bone alteration have the potential to improve the early diagnosis of OA, monitor OA progression, and evaluate treatment response.

Recently, several imaging modalities have been used to quantitatively assess subchondral microstructure. Radiography-based bone structure assessment has been performed using density measurement, fractal signature analysis, and trabecular microstructure analysis [8–10]. The results demonstrate that structural or density changes in the subchondral bone are associated with the onset and progression of OA. Some texture features-based models on X-rays have shown values in the prediction of knee OA and bone fragility assessment [11–13]. However, a two-dimensional (2D) plain radiograph is a projection of a three-dimensional (3D) structure, which lacks information on other joint structures involved in the disease progression. Compared to radiography, magnetic resonance imaging (MRI) is considered the optimal imaging modality for OA assessment due to its ability to produce a 3D structure and simultaneously image other tissues such as the cartilage, subchondral bone, and menisci [14]. Several magnetic resonance (MR) semi-quantitative scoring systems or quantitative methods of subchondral bone microstructure analysis have also been attempted, including trabecular morphological parameters, topological parameters, and texture analysis [15–17]. While the initial results are promising, there remain several issues: For example, the calculated morphological or topological parameters are highly sensitive to changes in image acquisition parameters, leading to poor reproducibility and limited generalizability. Although previous studies have shown that MRI texture features are significantly associated with ground-truth subchondral bone histomorphometry at the tibial plateau [18], the texture method involved is able to analyze a restricted number of slices and uses a limited number of features, and the predictive validity for early OA remains unclear.

Recently, radiomics was introduced to assess tissue and lesion characteristics. Compared to texture analysis, the radiomics method can provide more features based on images with different filtering without the limitation of requiring dedicated texture analysis programs, thus presenting greater possibilities for developing new image-based diagnostic biomarkers [19]. Radiomics is commonly used in clinical oncology for cancer detection, diagnosis, prognosis, and treatment response prediction [20]. However, the number of studies using this approach to investigate bone diseases is limited. Recently, some studies have evaluated MRI-based radiomics features for the assessment of knee OA. Hirvasniemi et al. [21] used MRI-based radiomics features from the tibial bone combined with machine learning to identify OA. However, the radiomics features were extracted only from the tibia. Given that more loading forces are concentrated in the two femoral condyles of the knee joint, we speculate that the constructed predictive model based on radiomics information extracted from the femoral condyle and combined with machine learning may improve diagnostic performance. Therefore, in this study, we aim to develop a novel magnetic resonance imaging (MRI)-based radiomics predictive model for the identification of knee OA based on the tibial and femoral subchondral bone and compared with the conventional trabecular structural parameter-based model.

## Materials and methods

### Participants

This retrospective study was approved by the institutional review board of Shanghai Ninth People’s Hospital (No.SH9H-2020-T395-2). Written informed consent was obtained from all participants. This was a cross-sectional study carried out at our institution between October 2020 and May 2021. Eighty-eight consecutive subjects were recruited by an orthopedic surgeon with 10 years of experience. The exclusion criterions were as follows: history of previous ipsilateral knee injury or surgery, inflammatory arthritis, osteonecrosis, metabolic bone disorder, and other diseases that affect the bone structure; MRI was contraindicated or with poor image quality. The heights and weights of the participants were recorded at the time of examination. All participants completed the standardized Western Ontario and McMaster Universities Arthritis Index questionnaire for pain, stiffness, and functional impairment to evaluate the severity of knee symptoms. In this study, we selected a sample size of 88, which is also similar to our previous feasibility studies for assessing subchondral bone [22].

### MRI acquisition

All participants were scanned using a 3 T MRI scanner (Achieva 3.0TX; Philips Healthcare, Best, Netherlands) with an eight-channel knee coil (Philips Healthcare). The knee flexion angle was adjusted to 20–30°, and an immobilization sponge was used to increase participant comfort and reduce motion artifacts. The MRI protocol included four sequences. A sagittal 3D balanced fast-field echo (3D BFFE) sequence (repetition time/time to echo [TR/TE] = 10/5.0, field of view [FOV] = 14 cm, matrix = 640 × 640, flip angle = 15°, in-plane spatial resolution = 0.234 mm × 0.234 mm, slice thickness = 1.5 mm, sensitivity encoding [SENSE] = 2, scan time = 8 min 14 s) was used to image the trabecular bone. A sagittal fat-suppressed 3D water-selective cartilage sequence (TR/TE = 20/6.0, FOV = 14 cm, matrix = 640 × 640, flip angle = 30°, in-plane spatial resolution 0.35 mm × 0.35 mm slice thickness = 1.5 mm, SENCE = 2, scan time = 5 min 40 s) was used to image articular cartilage. Sagittal and coronal 2D fast spin-echo proton density-weighted sequences with fat suppression were used to evaluate BMLs and osteophytes (TR/TE = 2500/30, FOV = 16 cm, matrix = 356 × 280, flip angle = 90°, in-plane spatial resolution 0.50 mm × 0.50 mm, section thickness = 3 mm).

### MRI assessment

Knee MRIs were scored by two board-certified radiologists using the MRI Osteoarthritis Knee Score (MOAKS) [23]. Two readers were extensively trained as described previously and blinded to the subjects’ clinical information [15]. Using MOAKS, the knee was divided into 14 subregions for scoring articular cartilage and BMLs. Cartilage loss was defined as MOAKS Grades 0–3 depending on size. A BML was defined as a hyperintensity on proton density-weighted imaging and graded as MOAKS Grades 0–3 depending on size by volume. For osteophyte scoring, each of the 12 locations was scored and graded according to size as follows: Grade 0, none; Grade 1, small; Grade 2, medium; and Grade 3, large. Each patient was given an overall cartilage loss, BML, and osteophyte score based on the most severe lesion in each of the subregions. Finally, the total score for each participant was calculated by adding the cartilage, BML, and osteophyte scores.

The identification of structural OA on MRI was based on previously proposed definition [24]. In brief, tibiofemoral OA was defined as the presence of definite osteophyte formation or full thickness cartilage loss or at least one of the following features: (1) subchondral bone marrow lesion or cyst not associated with meniscal or ligamentous attachments. (2) Partial thickness cartilage loss. (3) meniscal subluxation, maceration or degenerative tear [24]. Furthermore, the severity of OA was graded using the 5 Kellgren & Lawrence (KL) grades, mild OA was defined as KL (1–2) and advanced OA was KL (3–4) [25]. The normal cohort was defined as no knee pain and MOAKS grades 0.

### Segmentation of subchondral bone regions

**Fig. 1:**
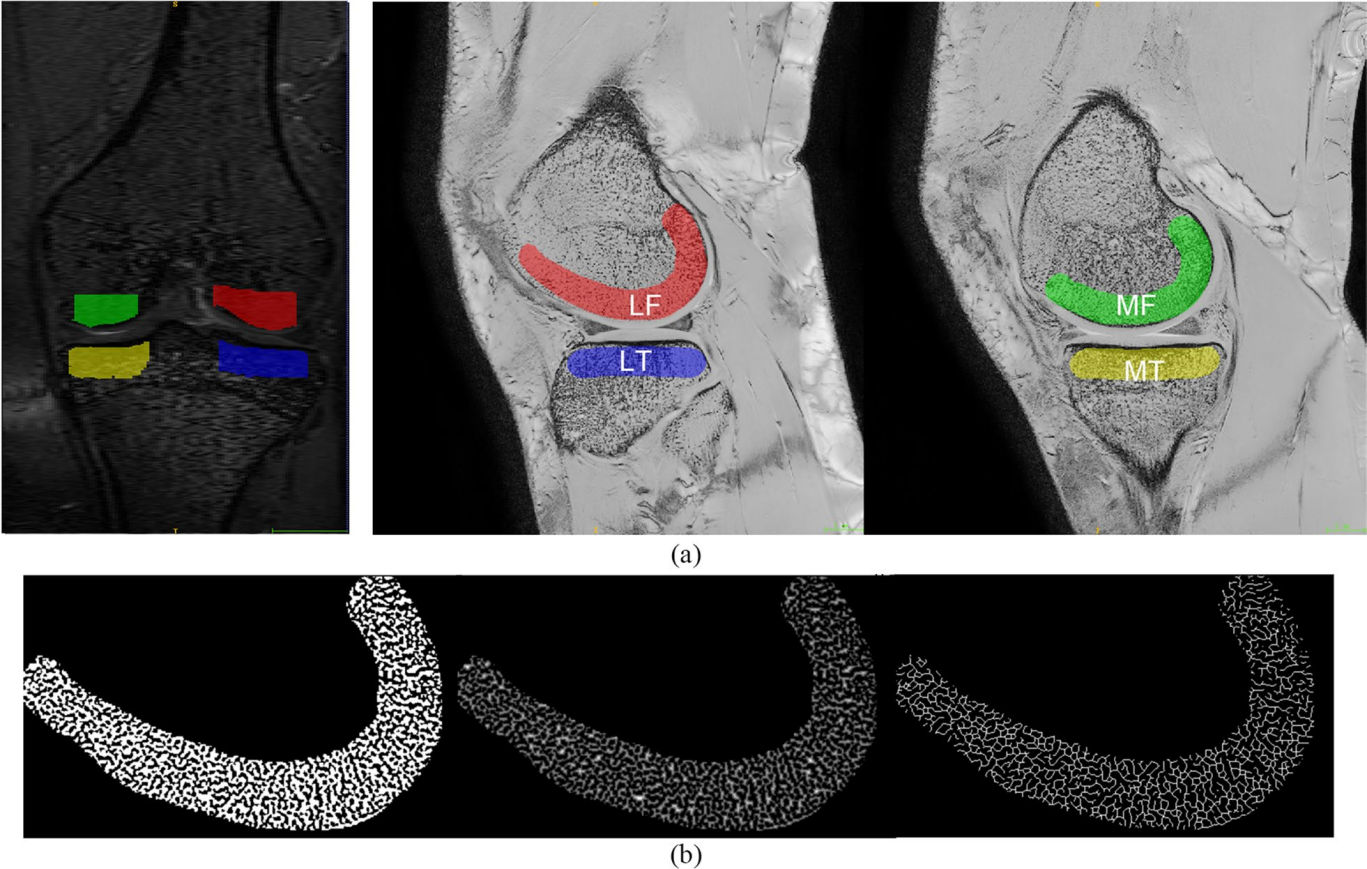
Region delineation and trabecular bone segmentation. **a** Four regions of interests (ROIs) were segmented from knee joint MR images named medial femoral condyle (MF), lateral femoral condyle (LF), medial tibial plateau (MT) and lateral tibial plateau (LT). **b** Obtained trabecular bone segmentation at the lateral femur. From left to right: binarized using adaptive threshold; after the distance transformation; after moving redundant pixels, which was obtained by multiplying the second one with the morphological skeletons

The 3D BFFE scans were used in the subchondral bone quantitative analyses. Four subchondral bone regions of the whole knee weight bearing articular surface were selected as regions of interest (ROIs): the medial femoral condyle (MF), lateral femoral condyle (LF), medial tibial plateau (MT), and lateral tibial plateau (LT) (Fig. 1a). We delineated 10 mm-wide band-like structure covered by cartilage in the sagittal image as the ROI (Fig. 1a). Note that, ROIs were segmented with the aid of 2D U-net convolutional neural network [26] that previous studies demonstrated its efficacy and precision in quickly generating segmentation [27]. To train the network, we firstly delineated the ROIs in 20 samples beforehand, then selected 100 2D slices from each images to get hundreds of images as training data to construct the U-net model. Then, we used it to segment the ROIs for the remaining images. The automatically acquired segmentations were manually revised by a skilled radiologist (CL) using ITK-SNAP, a free open-source software tool (www.itk-snap.org).

### Subchondral structural parameters measurements

For trabecular structural assessment, four trabecular morphological parameters, trabecular thickness (Tb.Th), trabecular separation (Tb.Sp), bone volume/total volume (BV/TV), and trabecular number (Tb.N), were measured according to the method of Sell et al*.* [28]. The ROIs were binarized using local adaptive thresholding to segment the image into trabeculae and marrow following the previous study [29]. The local thickness was calculated using distance transformation [30], which was assigned to each pixel in the trabeculae as the Euclidean distance from the pixel to the nearest pixel of the marrow. Redundant pixels were then removed by skeletonizing the trabeculae to produce morphological skeletons. Therefore, the overall mean width of the trabeculae Tb.Th was obtained by using the average of all pixels in the skeletons. Similarly, Tb.Sp was calculated by applying the same method to the marrow pixels. Figure 1b shows the trabecular structures obtained from the MR images. All image-processing processes were implemented using *skimage* [31] in Python (https://scikit-image.org/).

### Radiomic features extraction

Radiomic features of the subchondral bone were extracted from the four ROIs with no filtering (original image) and with Laplacian of Gaussian (LoG) filtering (*σ* = 1.0, 1.5, 2.0, and 2.5 mm). The extracted first-order and texture features included six classes: 1) first-order statistics (*n* = 18), 2) gray-level co-occurrence matrix (*n* = 24), 3) gray-level run length matrix (*n* = 16), 4) gray-level size zone matrix (*n* = 16), 5) neighboring gray-tone difference matrix (*n* = 5), and 6) gray-level dependence matrix (*n* = 14), totaling 93 features (reported in Additional file 2: Table S1). The extraction was implemented using *PyRadiomics* (https://pyradiomics.readthedocs.io/en/latest/), a toolkit recently developed in Python for the standardized automatic extraction of radiomics features [32].

### Feature selection and model construction

Before constructing the classification model, redundant features should be eliminated to reduce computation complexity and prevent overfitting issues. Hence, we conducted feature selection by applying the least absolute shrinkage and selection operator (LASSO) [33]. The feature values were z-scores standardized prior to the feature selection. Generally, LASSO introduces a penalty term that is equal to the absolute sum of regression coefficients. Depending on the penalty term, LASSO minimizes all regression coefficients toward zero and makes the coefficients zero for irrelevant features. To optimize the penalty parameter of LASSO, we performed five-fold cross-validation with 10,000 iterations. We then investigated whether the selected features significantly differed between positive and negative patients, and features with *P* < 0.05 were reserved. To prevent multicollinearity from resulting in inaccurate parameters for the final classification models, Pearson’s correlation coefficients were calculated after LASSO, and one of two features with Pearson’s coefficients of *r* > 0.90 was eliminated.

**Fig. 2:**
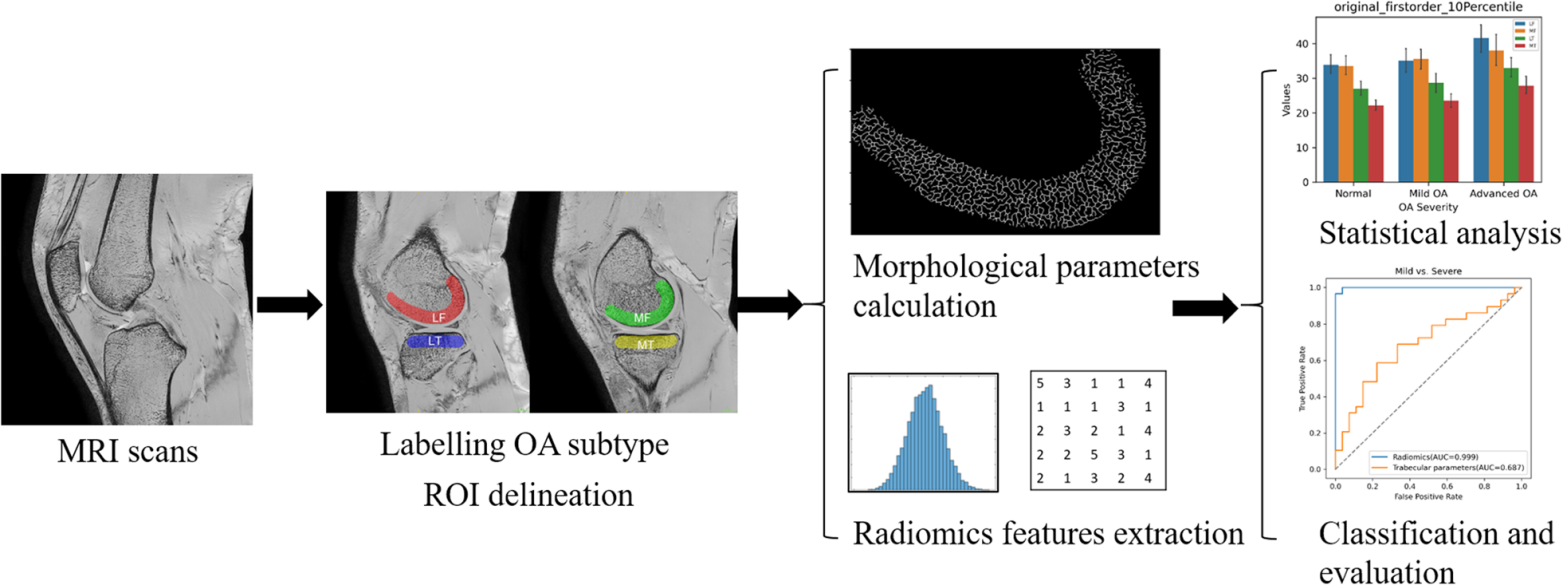
The workflow used in this study. From left to right: MRI scans (3D BFFE sequences for subchondral bone); Labeling the subjects with different severities of OA, and delineating the four regions of interest; Calculating the structural parameters and radiomics features; Performing statistical analysis, constructing and evaluating the models

Subsequently, four support vector machine (SVM) models were established to classify normal vs. OA, normal vs. mild OA, mild OA vs. advanced OA, and normal vs. advanced OA using the selected radiomics features. Different kernel functions of SVM were tested to find the best-performing model, including linear, radial basis function (RBF), cubic, and sigmoid kernel functions. The models were then trained and validated using fivefold cross-validation method. To show the relative merits of radiomics features compared with trabecular parameters, we constructed models using all four trabecular parameters to classify OA. The process of our study is schematically summarized in Fig. 2.

### Statistical analysis

Categorical data were compared using the chi-square test, while age and body mass index (BMI) were compared using one-way analysis of variance (ANOVA). For structural parameters and radiomics features, descriptive statistics were calculated, and differences in each indicator between the four regions were also analyzed using analysis of covariance (ANCOVA), taking the significant demographic characteristics as covariates. The covariates were also incorporated as predictors in the model. Differences in radiomics features between the two groups were investigated using the Mann–Whitney *U* test. Differences were considered significant at the two-sided *P* < 0.05. Pearson’s correlation coefficients for features and trabecular parameters were obtained using null hypothesis significance testing, and significance was set at *P* < 0.05. When evaluating the diagnostic performance of SVM models, receiver operating characteristic (ROC) curves were plotted, and the area under the curve (AUC) was calculated along with its 95% confidence intervals (CIs). Other indicators of accuracy, sensitivity, specificity, and the F1 score were also used to assess the predictive power of the models.

## Results

### Participant characteristics

Demographic characteristics of the 88 patients are summarized in Table 1. According to MOAKS scores and KL grades, 32 had no OA, 27 had mild OA, and 29 had advanced OA. The intra- and interobserver reliability results have been previously published [15, 34]. There was significant difference in age among the three groups (*P* < 0.001), whereas sex, BMI, and whether the left or right knee was affected were no significant differences among the three groups (*P* > 0.05). Therefore, we included age in our models as a predictor of OA.

**Table 1:** Subjects demographic and clinical characteristics

Variables	Total (*n* = 88)	Normal (*n* = 32)	Mild OA (*n* = 27)	Advanced OA (*n* = 29)	*P* value
Age(years)	52.2 ± 15.3	37.2 ± 9.4	56.5 ± 9.9	64.7 ± 9.9	< 0.001
Gender					0.44
Male	39(44.3%)	17((53.1%)	11(40.7%)	11(37.9%)	
Female	49(55.7%)	15(46.9%)	16(59.3%)	18(62.1%)	
BMI	24.6 ± 3.1	23.8 ± 2.6	24.9 ± 3.5	25.3 ± 2.8	0.13
Knee					0.66
Left knee	43(48.8%)	18(56.2%)	12(44.4%)	15(51.7%)	
Right knee	45(51.2%)	14(43.8%)	15(55.6%)	14(48.3%)	
MOKAS	3.44 ± 3.21	0.00	3.30 ± 1.13	7.38 ± 1.17	< 0.001

*BMI* Body mass index, *MOKAS* MRI Osteoarthritis Knee Score

### Morphological parameter analysis

Four trabecular structural parameters were obtained per subject. The reproducibility for the trabecular structural parameter has been previously published [35]. The mean values and standard deviations of each parameter are presented in Table 2. From the results, in OA patients, not only the tibial plateau but also the femoral condyle had a higher BV/TV than those of the normal cohort. Also, higher Tb.Th was present at the plateau (*P* < 0.05). Similar differences were also observed between patients with mild and advanced OA.

**Table 2:** Subchondral structural parameters in the femoral condyle and tibia plateau

	Normal (n = 32)	Mild OA (n = 27)	Advanced OA (n = 29)
*Lateral femoral condyle*			
BV/TV	0.269 ± 0.005	0.269 ± 0.005	0.273 ± 0.006 ^a^ ^b^
Tb.Th	0.166 ± 0.004	0.166 ± 0.004	0.168 ± 0.004
Tb.Sp	0.452 ± 0.008	0.452 ± 0.008	0.448 ± 0.013
Tb.N	1.615 ± 0.031	1.615 ± 0.030	1.621 ± 0.040
*Medial femoral condyle*			
BV/TV	0.269 ± 0.006	0.269 ± 0.005	0.273 ± 0.005 ^a^ ^b^
Tb.Th	0.162 ± 0.004	0.163 ± 0.004	0.164 ± 0.003
Tb.Sp	0.442 ± 0.010	0.444 ± 0.010	0.437 ± 0.009 ^a^ ^b^
Tb.N	1.652 ± 0.032	1.644 ± 0.034	1.662 ± 0.029
*Lateral tibia plateau*			
BV/TV	0.266 ± 0.006	0.267 ± 0.005	0.268 ± 0.006
Tb.Th	0.162 ± 0.004	0.164 ± 0.006	0.166 ± 0.005 ^b^
Tb.Sp	0.449 ± 0.009	0.451 ± 0.011	0.452 ± 0.009
Tb.N	1.633 ± 0.029	1.625 ± 0.042	1.617 ± 0.033
*Medial tibia plateau*			
BV/TV	0.264 ± 0.006	0.264 ± 0.005	0.269 ± 0.005 ^a^ ^b^
Tb.Th	0.159 ± 0.004	0.159 ± 0.004	0.163 ± 0.004 ^a^ ^b^
Tb.Sp	0.443 ± 0.011	0.442 ± 0.009	0.442 ± 0.008
Tb.N	1.660 ± 0.035	1.664 ± 0.035	1.651 ± 0.027

Data are mean ± standard deviation. The significance between groups is shown based on ANCOVA with adjustment for age

^a^ *P* < 0.05 in comparison with control subjects

^b^ *P* < 0.05 in comparison between mild OA and advanced OA

### Radiomic features and correlation analysis

A total of 93 features from each ROI were extracted from the MR images. Twenty-nine features were selected for normal vs. OA (LF, 6 features; MF, 8 features; LT, 4 features; and MT, 11 features), whereas 31 features were selected for mild OA vs. severe OA (LF, 11 features; MF, 10 features; LT, 3 features; and MT, 7 features). In normal vs. severe OA, 28 features were present (LF, 7; MF, 12; LT, 3; and MT, 6), and when classifying knees with and without mild OA, 13 features were selected (LF, 4; MF, 5; LT, 2; and MT, 2).

**Fig. 3:**
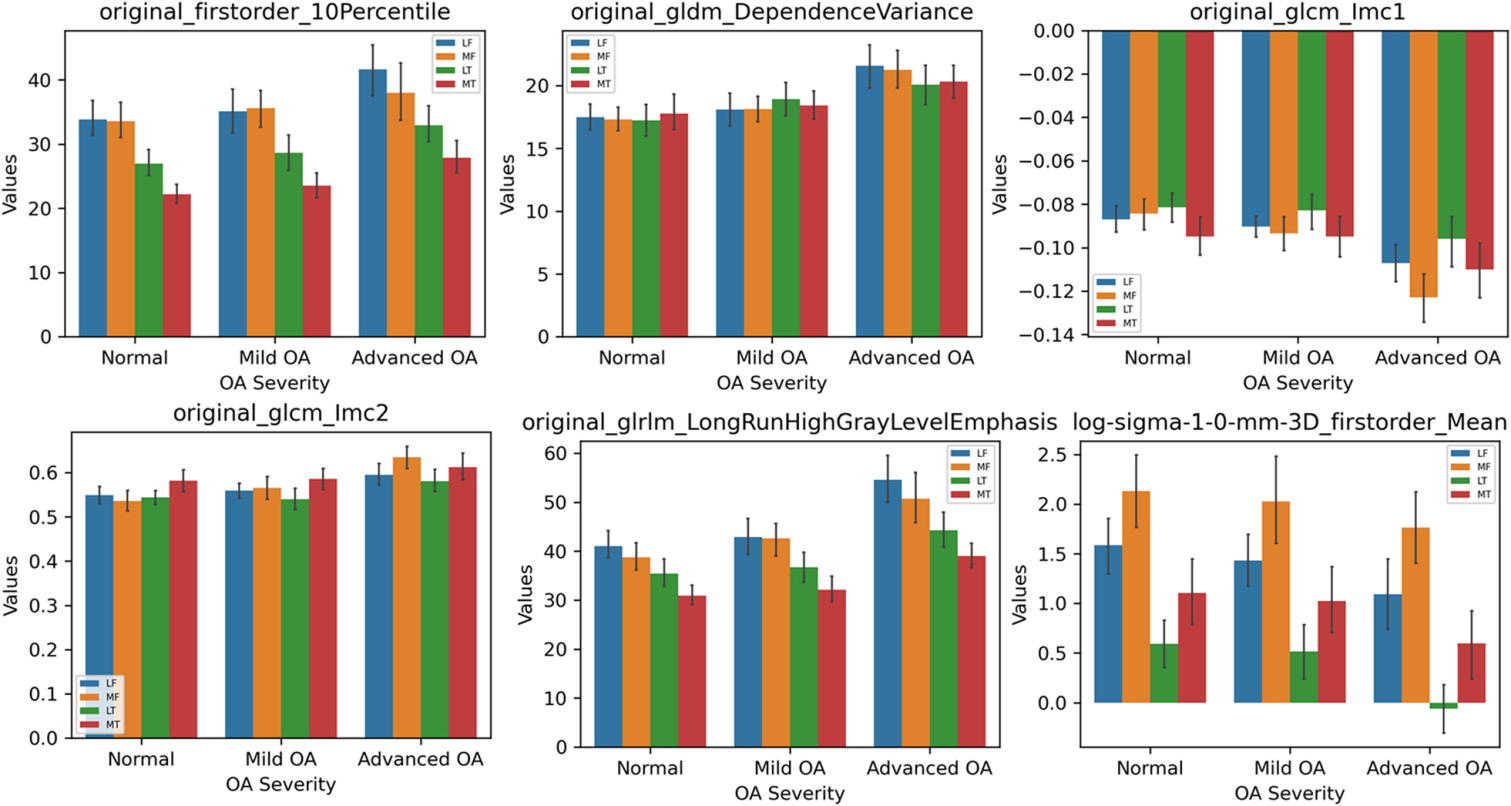
Comparison of six radiomics features between normal, mild OA and advanced OA groups in the four regions. Error bars represent the 95% confidence interval

Attention should be paid to the differences in radiomics features between both different groups and regions. Figure 3 compares the data distributions of six radiomics features from different groups and regions. In different ROIs with the same OA severity, some first-order features (e.g., 10th percentile of intensity), high-order texture features (e.g., Long Run High Gray-Level Emphasis), and features from LoG-filtered images (e.g., mean intensity) demonstrated significant differences, whereas these features differed between OA severities’ groups in the same regions.

Additionally, the selected features showed a correlation with the four trabecular structural parameters calculated from the MR images. To show the strength and direction of correlation statistically, features with a moderate correlation (*r* > 0.4, *P* < 0.05) with at least one parameter are listed in Table 3.

**Table 3:** Pearson’s correlation coefficients between radiomics features and trabecular bone parameters in four regions

ROI	Filter	Feature	Tb.Th	Tb.Sp	BV/TV	TbN
LF	original	glcm_Imc1	0.420**	0.006	−0.386**	0.146
MF	original	glcm_Imc1	− 0.412**	0.151	− 0.473**	0.015
	original	gldm_DependenceVariance	0.245*	−0.589**	0.665**	0.418**
	LoG(*σ*1.0 mm)	glrlm_RunVariance	0.131	−0.453**	0.463**	0.342**
LT	original	gldm_DependenceVariance	0.557**	−0.140	0.722**	−0.132
	original	glrlm_LongRunHighGrayLevelEmphasis	0.669**	0.324**	0.519**	−0.517
	LoG(*σ*1.0 mm)	firstorder_Mean	−0.577**	−0.429**	−0.342**	0.553**
MT	original	glcm_MaximumProbability	0.370**	−0.537**	0.712**	0.292**
	original	gldm_DependenceVariance	0.463**	− 0.524**	0.789**	0.243*
	original	gldm_LargeDependenceHighGrayLevelEmphasis	0.671**	0.049	0.585**	−0.312**
	original	glszm_SmallAreaLowGrayLevelEmphasis	0.171	−0.376**	0.421**	0.236*
	LoG(*σ*1.0 mm)	firstorder_Median	− 0.584**	− 0.219*	−0.390**	0.415**
	LoG(*σ*1.5 mm)	firstorder_Median	− 0.489**	− 0.273**	−0.268*	0.421**

*LF* Lateral femoral condyle, *MF* Medial femoral condyle, *LT* Lateral tibial plateau, *MT* Medial tibial plateau. Significant correlations are highlighted with asterisks (* or **). *P < 0.05; **P < 0.01

### Prediction performance evaluation

**Fig. 4:**
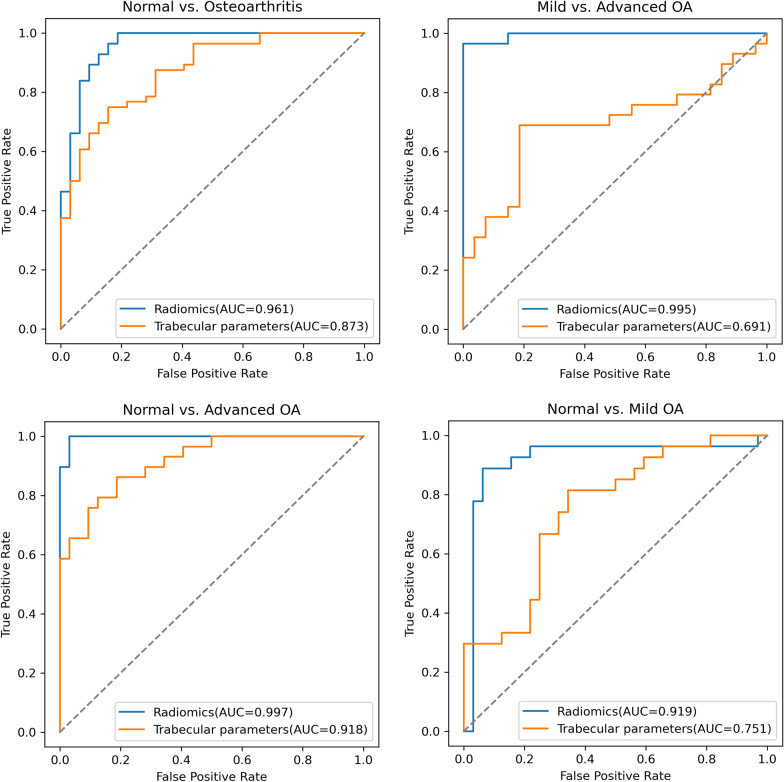
The receiver operating characteristic (ROC) curves of normal vs. OA, mild vs. advanced OA, normal vs. advanced OA and normal vs. mild OA

The SVM models based on the selected features successfully classified patients with and without OA. The comparison of different kernel functions shows that the RBF kernel is most suitable for the prediction of OA (seen in Additional file 1: Fig. S1). As observed in Fig. 4, the AUC was 0.961 (95% CI, 0.912–1.000) in classifying normal vs. OA, with the model combining radiomics features extracted from all four regions. The AUC was 0.873 (95% CI, 0.788–0.957), which was obtained using trabecular parameters as model variables. Similar results were observed for the other three classifications. The AUC was 0.995 (95% CI, 0.975–1.000) in classifying mild vs. severe OA, 0.997 (0.983–1.000) in classifying normal vs. severe OA and 0.919 (0.847–0.991) in classifying normal vs. mild OA, which were all higher than those when using trabecular parameters (Table 4, Fig. 4). The obtained AUCs were higher for classifying advanced OA than that for mild OA.

**Table 4:** AUC, accuracy, sensitivity, specificity and F1 score of four classification cases obtained from the radiomics-based and trabecular parameters-based model

	Normal vs. OA		Mild vs. advanced OA		Normal vs. advanced OA		Normal vs. mild OA	
	Radiomics	Trabecular	Radiomics	Trabecular	Radiomics	Trabecular	Radiomics	Trabecular
AUC	0.961 (0.912–1.000)	0.873 (0.788–0.957)	0.995 (0.975–1.000)	0.691 (0.551–0.830)	0.997 (0.982–1.000)	0.918 (0.846–0.990)	0.919 (0.847–0.995)	0.751 (0.628–0.874)
Accuracy	0.920	0.765	0.964	0.625	0.984	0.820	0.898	0.727
Sensitivity	0.982	0.911	0.966	0.690	1.000	0.828	0.889	0.518
Specificity	0.812	0.562	0.963	0.556	0.969	0.812	0.906	0.750
F1 score	0.919	0.774	0.964	0.623	0.984	0.820	0.898	0.639

*AUC* Area under the curve. AUCs were given with the 95% confidence interval

## Discussion

In this study, we constructed a predictive model based on MRI radiomics features extracted from the subchondral bone of the femur and tibia to identify OA. The results showed that multi-ROI radiomics-based models have a high AUC for distinguishing knee OA. The high diagnostic performance indicated that the radiomics-based model of subchondral bone has the potential to be a powerful tool for discerning knee OA, and it is more sensitive than models based on trabecular morphological parameters.

Emerging evidence suggests that deterioration of the subchondral bone microstructure could alter the stress distribution and load absorption of cartilage, and this is thought to cause physical damage [3]. In this study, our results showed that patients with OA of the MT had a higher BV/TV and thickened trabecular bone than normal in the tibial plateau. This was consistent with a previous study describing alterations in the subchondral bone [36, 37]. We attribute it that the majority of participants had predominantly medial compartment disease, and the medial tibia had better biomechanics [38]. Similarly, in the medial femur, higher BV/TV and lower Tb.Sp were observed in advanced OA. In the lateral femur, the BV/TV was also higher in patients with OA. These findings indicate that trabecular structural parameters could potentially reflect subchondral bone sclerosis, and that these parameters may be biomarkers for OA progression. Therefore, we constructed a predictive model based on structural parameters to identify OA, but the diagnostic performance was not sufficient (AUC 0.751 in normal vs. mild OA). This may be attributed to the limited spatial resolution of MRI and image binarization using arbitrary thresholds.

To improve diagnostic performance, we performed 3D radiomics analysis of the subchondral bone structure. Radiomics analysis showed that knees with OA had more heterogeneous and less spatially organized subchondral bone than those without. In our study, more advanced OA was associated with a higher dependence variance, indicating greater heterogeneity. Lower informational measure of correlation 1 and higher informational measure of correlation 2 (definitions can be found on *PyRadiomics*) showed lower complexity (or more uniformity) of the intensity within the regions of subchondral bone, which is consistent with a previous texture analysis based on T1-weighted sequences [39]. Radiomics features from LoG-filtered images also play an important role in distinguishing OA severity, providing more information than when using texture analysis on the same image. As an edge detection operator, LoG enhances intensity changes in images and thus can reflect structural changes in the bone. Radiomics analysis also demonstrated that higher OA severity levels had lower mean intensity values in the LoG-filtered images.

Based on radiomics features, we obtained a higher diagnostic performance for classifying OA than when using models based on trabecular structural parameters (AUC 0.961 vs.0.873). These results were also confirmed by MacKay et al. [40] that texture analysis outperforms trabecular structural analysis when distinguishing OA patients from healthy controls. Compared with the study of Hirvasniemi J. et al.[21], our predictive model yielded a relatively higher discrimination ability for OA (AUC, 0.961 vs. 0.800), implying that multi-ROI radiomics feature analysis can make improvements for the identification of knee OA.

Furthermore, we investigated the correlation between selected radiomics features and trabecular structural parameters to find more possible interpretations of the radiomics features. Calculating morphological structural parameters from the ROIs in MR images is more practical and sufficiently reliable compared to histomorphometry. Pearson’s correlation coefficients demonstrated that several radiomics features were strongly correlated with trabecular parameters (*r* > 0.6, *P* < 0.05), suggesting that changes in the microstructure of subchondral bone may reflect the multidimensional radiomics features of MR images. For example, increased dependence variance within the lateral tibia and medial femur were strongly associated with higher Tb.Th, BV/TV, and Tb.N, but with lower Tb.Sp. These changes represent a trabecular structural alteration in the progression of OA.

This study had several limitations. First, the number of patients was limited, which might have introduced more stochastic effects. Second, this study was monocentric, and the generalizability of our model requires further investigation in multicenter MRI datasets with larger sample sizes. Third, the 3D BFFE sequences used in this study are not commonly available for routine use due to their longer acquisition time, leading to limited generalizability. Finally, the inclusion of clinical risk factors and changes in the cartilage may improve the model’s diagnostic performance for assessing the severity of knee OA.

## Conclusions

Radiomics features extracted from both the femoral and tibial subchondral bone revealed differences between knees with and without OA, which can be used as quantitative biomarkers for OA in the future. MRI-based multi-ROI radiomics features may help investigate OA-related microstructural changes.

## Supplementary Information

**Additional file 1: Fig. S1.** Comparison of four kernel functions (linear, RBF, cubic,and sigmoid) of SVM. Accuracy, AUC, sensitivity, and specificity are compared toshow the superiority of the RBF kernel in our experiments.

**Additional file 2 : Table S1.** Extracted radiomics features

## Data Availability

Data under study are available on request from the corresponding author, which are not publicly available due to privacy or ethical restrictions.
