# Supplementary figures and images for: Radiomics analysis using MR imaging of subchondral bone for identification of knee osteoarthritis
Source: J Orthop Surg Res. 2022 Sep 14;17:414. doi: 10.1186/s13018-022-03314-y (PMC9476345; doi:10.1186/s13018-022-03314-y)

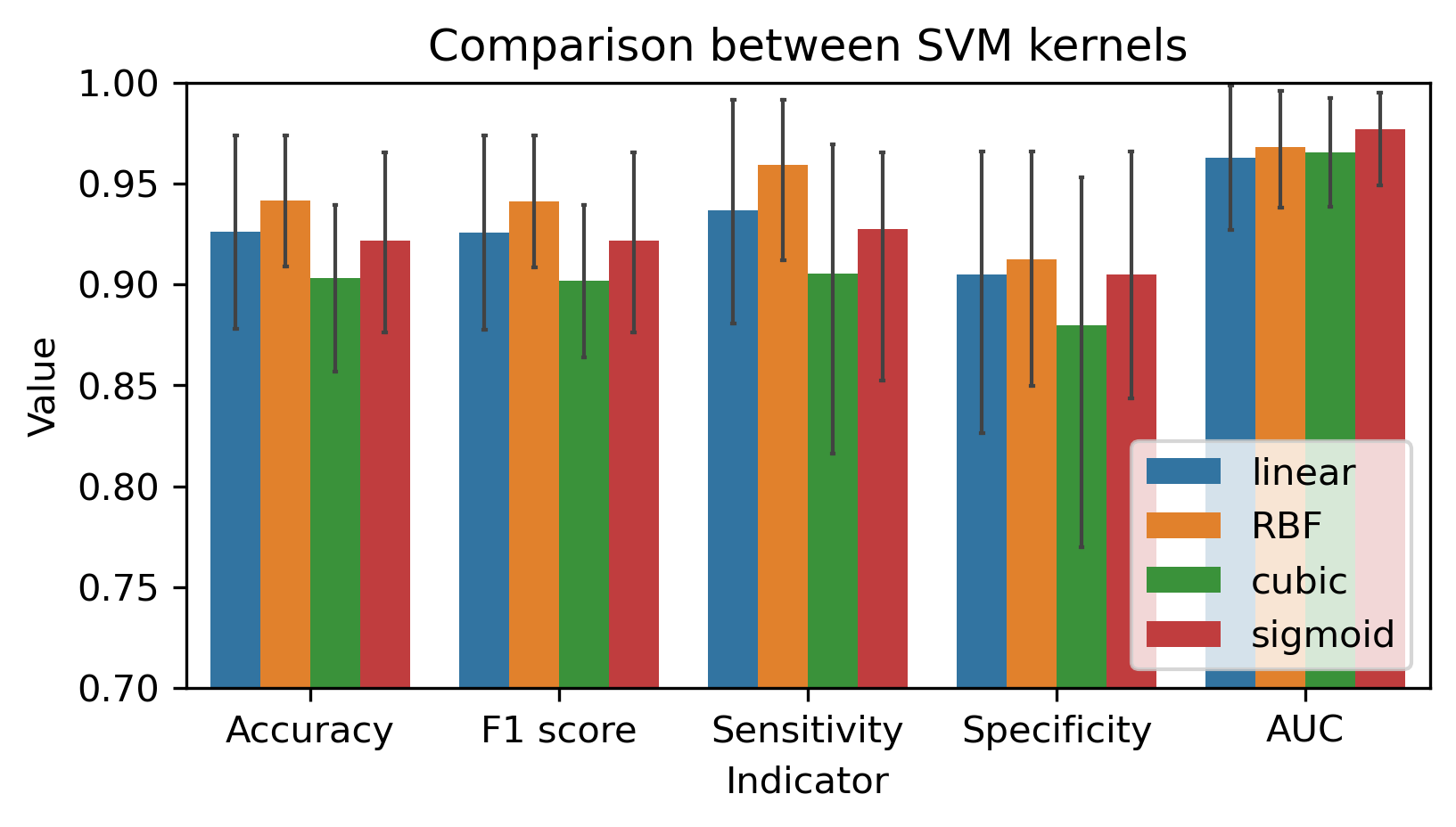

Supplement: Supplementary file 1 — Additional file 1: Fig. S1. Comparison of four kernel functions (linear, RBF, cubic,and sigmoid) of SVM. Accuracy, AUC, sensitivity, and specificity are compared toshow the superiority of the RBF kernel in our experiments. [file 13018_2022_3314_MOESM1_ESM.tif]
